# SH2B3 inactivation through CN-LOH 12q is uniquely associated with B-cell precursor ALL with iAMP21 or other chromosome 21 gain

**DOI:** 10.1038/s41375-019-0412-1

**Published:** 2019-02-28

**Authors:** Paul B. Sinclair, Sarra Ryan, Matthew Bashton, Shaun Hollern, Rebecca Hanna, Marian Case, Edward C. Schwalbe, Claire J. Schwab, Ruth E. Cranston, Brian D. Young, Julie A. E. Irving, Ajay J. Vora, Anthony V. Moorman, Christine J. Harrison

**Affiliations:** 1 Wolfson Childhood Cancer Research Centre, Northern Institute for Cancer Research, Newcastle University, Newcastle-upon-Tyne, UK; 2 Faculty of Health and Life Sciences, Northumbria University, Newcastle upon Tyne, UK; 3 Barts Cancer Institute, Queen Mary University of London, London, UK; 4 Great Ormond Street Hospital for Children NHS trust, London, UK

**Keywords:** Acute lymphocytic leukaemia, Cancer genomics, Genetics research, Oncogenes

## Abstract

In more than 30% of B-cell precursor acute lymphoblastic leukaemia (B-ALL), chromosome 21 sequence is overrepresented through aneuploidy or structural rearrangements, exemplified by intrachromosomal amplification of chromosome 21 (iAMP21). Although frequent, the mechanisms by which these abnormalities promote B-ALL remain obscure. Intriguingly, we found copy number neutral loss of heterozygosity (CN-LOH) of 12q was recurrent in iAMP21-ALL, but never observed in B-ALL without some form of chromosome 21 gain. As a consequence of CN-LOH 12q, mutations or deletions of the adaptor protein, *SH2B3*, were converted to homozygosity. In patients without CN-LOH 12q, bi-allelic abnormalities of *SH2B3* occurred, but only in iAMP21-ALL, giving an overall incidence of 18% in this sub-type. Review of published data confirmed a tight association between overrepresentation of chromosome 21 and both CN-LOH 12q and *SH2B3* abnormalities in B-ALL. Despite relatively small patient numbers, preliminary analysis linked 12q abnormalities to poor outcome in iAMP21-ALL (*p* = 0.03). Homology modelling of a leukaemia-associated SH2 domain mutation and in vitro analysis of patient-derived xenograft cells implicated the JAK/STAT pathway as one likely target for SH2B3 tumour suppressor activity in iAMP21-ALL.

## Introduction

Intrachromosomal amplification of chromosome 21 (iAMP21-ALL) defines a distinct, high-risk sub-group comprising 2% of precursor B-lineage acute lymphoblastic leukaemia (B-ALL) [1, 2]. Originating through breakage-fusion-bridge cycles and chromothripsis, rearrangements of chromosome 21, giving rise to iAMP21, although chaotic, result in common regions of genomic amplification and increased gene expression [3, 4]. The precise mechanism by which iAMP21 promotes leukaemia is currently unknown, but is assumed to involve increased expression of genes or functional non-coding sequences within the chromosome 21 amplified regions. Other structural amplifications of chromosome 21 (OSA21), such as isochromosome 21, i(21q), are rare but gains of one or more copies of whole chromosome 21 (wc21) are extremely common. Notably chromosome 21 is the only chromosome always overrepresented in high hyperdiploid, hypodiploid and near-haploid ALL [5, 6], which together account for about 30% of B-ALL. Somatically acquired trisomy or tetrasomy 21 may also be present as sole aneuploidies [7] and, individuals with constitutional trisomy 21 (Down syndrome, DS) are at an ~20-fold increased risk of developing B-ALL [8, 9].

Deletions of *RB1* and *EBF1*, mutations affecting the *RAS* pathway, *CRLF2* activating rearrangements, gain of the X chromosome and partial deletion of chromosome 7 are all enriched in iAMP21-ALL [4, 10–12]. Suggesting a mechanistic link between iAMP21 and overrepresentation of whole chromosome 21, some of these secondary abnormalities also occur at high frequency in hyperdiploid or DS-ALL [13, 14].

Duplication of part of the maternal or paternal genome with concurrent loss of equivalent sequence from the other parental chromosome, known as copy number neutral loss of heterozygosity (CN-LOH), can have pathological consequences, through imbalance of expression of imprinted loci or conversion to homozygosity of inherited or acquired mutations and copy number abnormalities (CNA). In B-ALL, only deletions of the *CDKN2A/B* genes have been clearly linked to CN-LOH 9p [15–20], but other regions were recurrent, suggesting that they harbour imprinted or mutated genes or CNA that contribute to ALL. Here, we investigated CN-LOH in B-ALL with whole or partial gain of chromosome 21. We uncovered a highly specific association between CN-LOH 12q and the presence of iAMP21/OSA21, or less frequently gain of wc21. We further identified *SH2B3* abnormalities as a target of CN-LOH 12q and found that, with rare exceptions, previously published cases of B-ALL with *SH2B3* abnormalities also harboured whole or partial amplification of chromosome 21. Preliminary analysis of outcome linked these 12q abnormalities to an increased risk of relapse in iAMP21-ALL.

## Materials and methods

### Patient and PDX material

Bone marrow or peripheral blood diagnostic, matched remission and/or relapse samples were obtained from patients in the United Kingdom, Germany or the USA. Ethical approval and consent was obtained for all patients in accordance with the declaration of Helsinki. Cytogenetic results were reviewed by the Leukaemia Research Cytogenetics Group [21]. PDX were generated from stored viable bone marrow cells from patients 78, 72, 62 and 69 as previously described [22]. Patient samples were obtained as DNA or viable cells from the reference laboratories or the Bloodwise Childhood Leukaemia Cell Bank, UK. DNA was extracted from viable patient cells or from blasts isolated from PDX spleens and purified over FICOL, using the DNeasy Extraction kit (Qiagen, Manchester, UK). Study specific IDs and references, for patients with whole or partial overrepresentation of chromosome 21, that have been previously reported [4, 10, 11, 22, 23] are indicated in supplementary table S1.

### Definition of chromosome 21 CN status

The majority of cases of iAMP21-ALL had been diagnosed on the basis of cytogenetic identification of an abnormal chromosome 21 and ≥3 additional RUNX1 FISH signals, with probes hybridising to the RUNX1 genomic region [24]. iAMP21 was confirmed in these cases from the characteristic chromosome 21 SNP 6.0 array profiles, as previously described [3]. In a minority of cases, where suitable material for cytogenetic or FISH analysis was unavailable or <3 extra copies of RUNX1 were present but flanking regions were more highly amplified, iAMP21 was identified by SNP 6.0 array CN profile alone (supplementary figure 4). Constitutional or somatically acquired aneuploidy 21 was identified by cytogenetic analysis and confirmed by SNP array as increased whole chromosome 21 CN. A minority of aneuploidies were defined on the basis chromosome 21 CN profile alone. Other cases, collectively referred to as other structural amplifications of 21 (OSA21), included those identified by cytogenetics as isochromosome 21q, i(21q), or isodicentric 21, idic(21), and less clearly defined abnormalities, which failed to meet our definitions of iAMP21 or aneuploidy. The techniques used to define chromosome 21 CN abnormalities are shown in Supplementary Table 1b for each case.

### Techniques described in the supplementary methods

Detailed methods for; SNP6.0 and methylation array analysis, Sequencing of *SH2B3*, homology modelling, western blots, STAT and ERK activation assays and statistical analysis are provided in the supplementary methods.

## Results

### The landscape of CN-LOH in B-ALL patients with whole or partial gain of chromosome 21

**Fig. 1:**
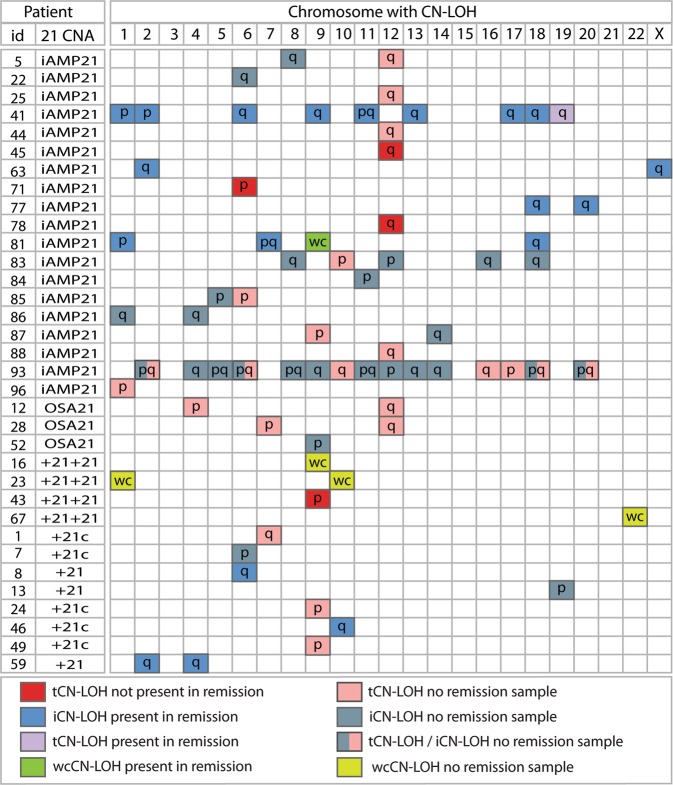
The landscape of CN-LOH in B-ALL with whole or partial overrepresentation of chromosome 21. Identification (ID) numbers and chromosome 21 CNA are shown in the left hand columns for patients carrying regions of CN-LOH identified by analysis of SNP 6.0 arrays. Chromosome numbers are indicated in the top panel and CN-LOH status for each patient by box colour as shown in the key. For patients with an available remission sample, the origin was defined as somatic (not present in remission) or germline (present in remission) with iCN-LOH always identified as germline and all but one tCN-LOH defined as somatic. Chromosomal position of CN-LOH is indicated by; p (segment/s of the short arm), q (segment/s of the long arm) pq (segments of both short and long arms but not the whole chromosome), wc (whole chromosome). Multiple regions of CN-LOH that were predominantly interstitial, seen in patients 41 and 93, are indicative of consanguinity. tCN-LOH 6p (*n* = 2), 9p (*n* = 4) and 12q (*n* = 8) and wcCN-LOH 9 (*n* = 2) were recurrent

**Fig. 2:**
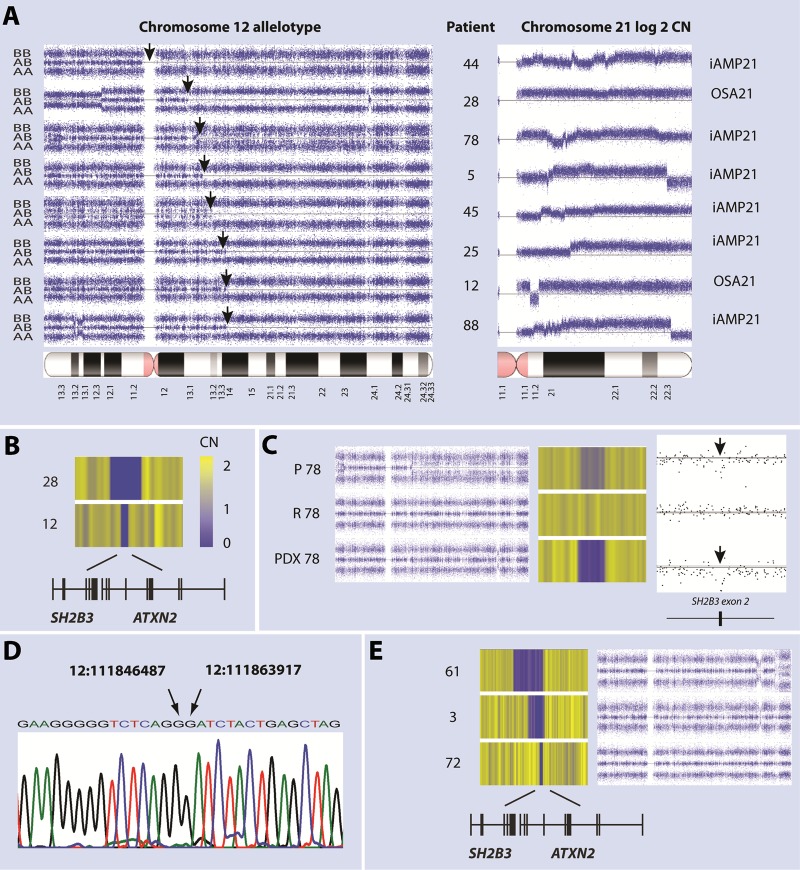
Co-occurrence of 12q CN-LOH, amplification of chromosome 21 and deletion of SH2B3. **a** On the left SNP 6.0 genotype profiles show the relative signal intensity of the B allele probes for chromosome 12. Normal genomic regions are characterized by three levels of intensity corresponding to the AA, AB and BB genotypes and regions of CN-LOH by loss of the AB genotype. Arrows mark the proximal boundary of CN-LOH which were terminal in each case. To the right are shown patient IDs and chromosome 21 log 2 CN profiles, black lines mark the mean signal intensity for a CN of 2. All patients showed evidence for structural amplification of chromosome 21, as indicated, six abnormalities were defined as iAMP21. **b** Heat maps of CN for a region of 12q showing clonal deletion in two patients with 12q CN-LOH. Both deletions were bi-allelic with the smaller of the two confined to exons of *SH2B3* and *ATXN2*. **c** Suggesting they target a common underlying genomic abnormality, allelotype and CN heat maps for a presentation sample from patient 78 (P 78) were consistent with the presence of competing sub-clones carrying 12q CN-LOH and a mono-allelic deletion that included the *SH2B3*/*ATXN2* region. In a PDX derived from the same sample (PDX 78), CN-LOH was lost while the deletion became clonal. To the right a detailed view of dot-plots of SNP 6.0 CN within the deleted region, suggests the presence of a somatically acquired bi-allelic micro-deletion centred on exon 2 of *SH2B3* in both patient and PDX samples but not the remission sample (R 78). Black lines indicate a copy number of 2. Arrows indicate the position of SH2B3 exon 2. **d** Sequence amplified from patient 78 using *SH2B3* intron 1 and 2 primers that defines breakpoints for a 17.4 Kb deletion. **e** SNP6.0 CN heat maps of deletions that included the *SH2B3* region found in three iAMP21 patients without CN-LOH 12q. In patient 61 the deletion is mono-allelic while in patients 3 and 72 nested deletions resulted in bi-allelic loss of *SH2B3*

From analysis of SNP6.0 arrays, we first determined the frequency and genomic location of CN-LOH in B-ALL patients with iAMP21-ALL (*n* = 49), OSA21-ALL (*n* = 11) and two (*n* = 5) or one (*n* = 34) extra entire copy of chromosome 21, either as a constitutional or acquired single aneuploidy or within a hyperdiploid or hypodiploid karyotype. Excluding two patients with multiple regions, that we attributed to consanguinity [25], five cases showed whole chromosome CN-LOH (wcCN-LOH), 27 involved interstitial regions (iCN-LOH) and 19 were terminal (tCN-LOH) (Fig. 1 and Supplementary Table 1a/b). In agreement with previous reports [16, 26], tCN-LOH were of somatic and iCN-LOH of germline origin as deduced from those cases with available matched, non-leukaemic samples. Notably, eight tCN-LOH involved the long arm of chromosome 12 (12q), six occurring in iAMP21-ALL and two in OSA21-ALL (Fig. 2a). Other recurrent regions involved the short arms of chromosomes 6 and 9 (6p, 9p) or whole chromosome 9 (wc9).

### tCN-LOH 12q is unique to a background of whole or partial amplification of chromosome 21

We then screened 203 B-ALL patients with normal chromosome 21 copy number (CN) and found tCN-LOH 9p in 10 cases but none involving 12q or 6p (Supplementary Table 2). To validate findings within our own institution, we investigated CN-LOH 12q and 6p among 974 patients from a broad range of B-ALL subtypes from six published studies (Table 1) [6, 15–17, 19, 20]. In patients with disclosed chromosome 21 CN status, 6p CN-LOH was associated with both partially amplified (*n* = 3) and apparently normal chromosomes 21 (*n* = 3). Among cases with CN-LOH 12q, one was described as iAMP21-ALL [17] and two co-occurred with amplifications of 21q to CN > 5 [15], thus were likely iAMP21-ALL. The remaining case harboured tetrasomy 21 [16]. So, taken together with our data, CN-LOH 12q was exclusive to a background of whole or partial chromosome 21 CN gain. The incidence, while only 0.9% among B-ALL patients overall, was 13% among our cohort of iAMP21/OSA21-ALL. The overall incidence of CN-LOH 6p was 1.1% and, not exclusive to, but possibly enriched in association with whole or partial gain of chromosome 21, as it occurred at a rate of 3% among our cases with and never without these abnormalities. A 70.3 Mb common region of 12q tCN-LOH was defined from our cases, with breakpoints clustered between the centromere and chromosome band 12q14 (Fig. 2a and Supplementary Table 1b).

**Table 1:** The incidence and location by cytogenetic band of tCN-LOH 12q and 6q identified in this study or previously published cases of B-ALL

Study	No. B-ALL patients	Patient ID	12q CN-LOH	6p CN-LOH	Chromosome 21 CN status
Present study	302	5	12q13.1-12qtel		iAMP21
		12	12q14-12qtel		OSA21
		25	12q14-12qtel		iAMP21
		28	12q12-12qtel		OSA21
		44	12q11-12qtel		iAMP21
		45	12q13.1-12qtel		iAMP21
		78	12q13.1-12qtel		iAMP21
		88	12q14-12qtel		iAMP21
		71		6ptel-6p22.1	iAMP21
		85		6ptel-6p21.2	iAMP21
Baughn 2015 [17]	65	Not reported	12q24.12-q24.32		iAMP21
Kawamata 2008 [15]	399	Not reported	12q? to 12qtel		21q CN > 5
		Not reported	12q? to 12qtel		21q CN > 5
		Not reported		6ptel-6p?	Normal
		Not reported		6ptel-6p?	21q CN > 5
		Not reported		6pter-6p?	21 CN 3–4
		Not reported		6pter-6p?	21 CN 3–4
Lundin 2016 [16]	181	28		6ptel-6p22.2	Normal
		31		6ptel-6p22.1	Unknown
		19	12q14.1-12qtel		+21,+21
Mullighan 2007 [20]	192	*TCF3-PBX1* #3		6ptel-6p22.1	Unknown
		*ETV6-RUNX1* #10		6ptel-6p21.1	Unknown
		HD 47-50 #22		6ptel-6p22.1	Unknown
		HD > 50 #34		6ptel-6p22.1	Unknown
		Pseudodiploid #8		6p12.3-6p21.31	Unknown
Paulsson 2010 [6]	116	HD > 50 (All)			
Ninomya 2012 [19]	21	AA214		6-p12.1-6pcen	Normal
		AA217		6p21.33-6p25.3	Normal

Position of CN-LOH and chromosome 21 CN reported by Kawamata [15] are estimated from Fig. 2a. HD (high hyperdiploid)

### CN-LOH 12q does not unmask an imprinted gene

To explore the possibility that CN-LOH caused unbalanced expression of an imprinted locus on 12q, we compared methylation array data from seven of the cases with CN-LOH and an equal number of iAMP21-ALL patients without evidence of a 12q abnormality. Specific genes; *DCN*, *WIF1*, *FBRSL1* and *E2F2*, had been predicted to be imprinted and fell within the 12q common region of CN-LOH (www.geneimprint.com/Catalogue _of Parent of Origen Effects). However no evidence was found for differential methylation, between the two groups, of probes associated with these genes (Supplementary Figure 1). Furthermore analysis of the entire 12q common region of CN-LOH revealed no significant blocks of differential methylation between cases with and without CN-LOH. Therefore we concluded that CN-LOH 12q was not driven by loss of imprinting in these cases.

### Deletions of the SH2B3 gene are coincident with CN-LOH 12q

We next investigated regions of CN-LOH 12q for coincident CNA and identified two with clonal deletions that focused attention on a region containing *SH2B3* and *ATXN2* (Fig. 2b and Supplementary table 3). In a third patient, and the patient-derived xenografts (PDX), sub-clones with 12q deletion or CN-LOH fluctuated in level, suggesting that competing sub-clones targeted a lesion on the second allele through different mechanisms. Close examination of presentation and PDX array data revealed evidence for an intragenic deletion of *SH2B3* centred on exon 2, not present in a remission sample (Fig. 2c). Loss of exon 2 was confirmed by PCR amplification of sequence from this sample, but not a normal control, using *SH2B3* intron 1 and 2 primers, which defined the breakpoints of a 17.4 Kb deletion (Fig. 2d). Albeit at low frequencies, mutations and CNAs of *SH2B3* have previously been described in B-ALL [27–30], so we further investigated this gene in iAMP21 and other sub-types of ALL.

### Deletions of *SH2B3* in the absence of CN-LOH are recurrent in iAMP21-ALL

We then analysed SNP6.0 arrays for 12q CN status in patients from our cohort with or without whole or partial chromosome 21 gain, without 12q CN-LOH. We identified a clonal mono-allelic and two bi-allelic deletions affecting *SH2B3* in three cases of iAMP21-ALL (Fig. 2e and Table S3). Deletion of *SH2B3* was never seen outside iAMP21-ALL. The combined incidence of 12q CN-LOH and/or deletion of the region including *SH2B3* was 9/49 (18%) in iAMP21-ALL, in marked contrast to non-iAMP21-ALL, where among 253 patients only two were seen (0.8%) and both cases carried OSA21. The association between amplification of chromosome 21 and CN-LOH/deletion 12q was highly significant in our cohort (*p* ≤ 0.01). To investigate a larger cohort with less bias towards chromosome 21 abnormalities, we screened publicly available SNP array data from 648 presentation B-ALL [28], identifying just two with *SH2B3* deletions, one with tetrasomy 21 and the other with no recorded evidence of a chromosome 21 CN abnormality (Supplementary Figure 2 and Supplementary table 4). These data confirmed that, in the absence of iAMP21/OSA21, deletion of the *SH2B3* region is extremely rare in B-ALL.

### Mutations of *SH2B3* are associated with CN-LOH and heterozygous deletion of 12q in iAMP21-ALL

**Fig. 3:**
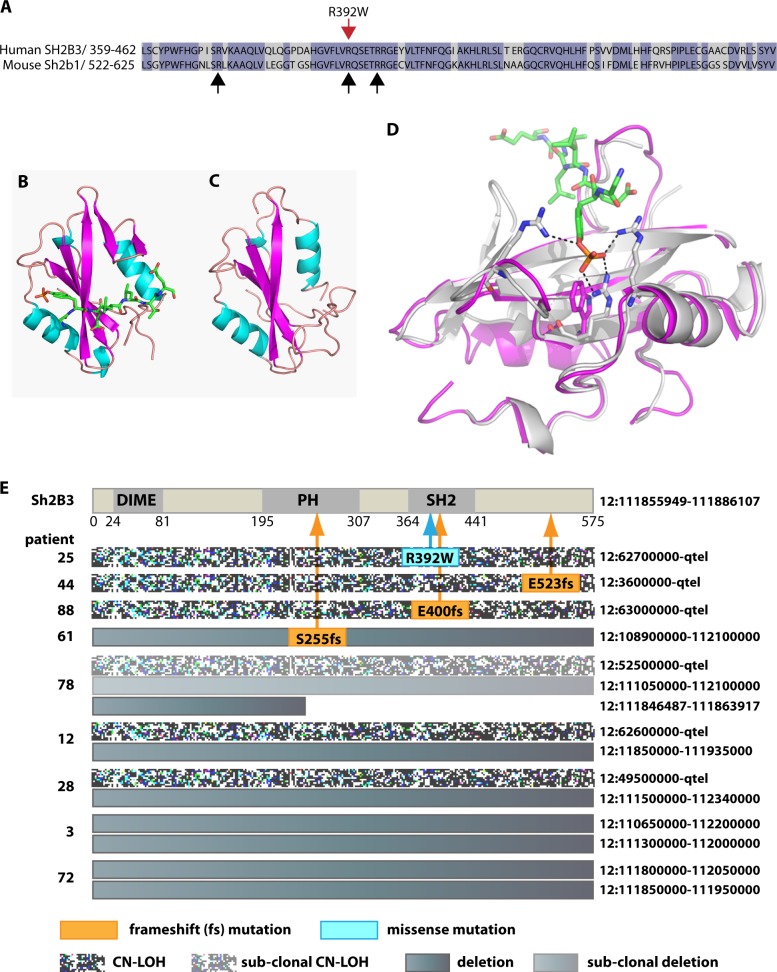
Impact of the R392W substitution and other SH2B3 abnormalities. **a** 104 amino acid sequence comprising 92% of the SH2 domain of human SH2B3 aligned to the mouse SH2B1 sequence. Black arrows indicate three arginine residues required to coordinate pTyr813 of JAK2 within the mouse SH2B1 SH2 binding pocket. Red arrow shows the position of the R392W arginine to tryptophan substitution identified in patient 25. **b** Ribbon diagram based on the published co-crystalized structure of the mouse SH2B1 SH2 domain and an eleven residue phosphopeptide surrounding tyrosine pTyr813 within the activation loop of JAK2 [40]. The structure has a canonical SH2 domain architecture with a central β-sheet (magenta) flanked by two α-helices (cyan) that form two binding pockets for the JAK2 phosphopeptide (shown as a stick representation with carbon atoms in green, oxygen atoms in red, nitrogen atoms in dark blue and phosphate atoms in orange). **c** A similar canonical SH2 domain structure is seen in a homology model of 104 residues of the SH2 domain of human SH2B3 based on the mouse SH2B1 crystal structure. Overall sequence identity between the two SH2 domains was 71%, with a structural homology of 1 Å RMSD when superposed. **d** Detailed view of superposed models of the phosphotyrosine binding pocket of SH2 domains of mouse SH2B1 (grey ribbons) and human SH2B3 (magenta ribbons) demonstrating close structural homology for this region. The JAK2 phosphopeptide and three arginine residues (R534, R555 and R560) that coordinate pTyr813 within the binding pocket through salt bridges (broken black lines) are indicated by stick representations, with carbon atoms of arginine in grey and other atoms coloured as in 3B. The R392W substitution in human SH2B3 is also shown as a stick representation with carbon atoms coloured in magenta. Tryptophan in this position is unable to form salt bridges with pTyr813, the R392W substitution therefore results in loss of two of four critical interactions with phosphotyrosine and is predicted to destabilise binding of SH2B3 to activated JAK. **e** Summary of abnormalities of SH2B3 identified in this study. At the top the SH2B3 linear protein structure is represented with major functional domains marked; DIME (dimerization), PH (Pleckstrin homology), SH2 (Src homology 2). For each patient, genomic abnormalities of the *SH2B3* coding region, as indicated in the key, are aligned with the domain representation. Numbers on the left are patient ids, genomic position of CN-LOH and deletion are indicated on the right. The codon position and consequence of somatically acquired mutations are as labelled, with arrows marking their positions on the SH2B3 protein. Through combinations of mutation, deletion and CN-LOH, *SH2B3* abnormalities were all homozygous and predicted to result in absence of a coding transcript (patients 3, 12, 28, 72 and 78), production of truncated and likely unstable transcripts / proteins (patients 44, 61 and 88) or production of a protein with functionally impaired SH2 domain (patient 25)

Although rare, mutations and deletions of *SH2B3* have previously been described in B-ALL [27–33], T-ALL [27, 34] and a range of myeloid neoplasms (reviewed in [35]). In addition, polymorphisms of *SH2B3* have been associated with skewed haematological parameters and predisposition to autoimmune disease or myeloid malignancy, the most common of these is rs3184504, which results in a R262W substitution that affects PH domain function [36, 37]. To investigate involvement of polymorphisms or acquired mutations, we carried out Illumina sequencing of coding exons of *SH2B3* in patients with CN-LOH and/or deletion of 12q (*n* = 9), as well as iAMP21-ALL patients without identified 12q abnormalities (*n* = 6) (Table 2). In four cases with bi-allelic deletion, as expected, no sequence abnormalities were found, but frameshift mutations accompanied 12q CN-LOH without deletion (*n* = 2) or mono-allelic deletion without 12q CN-LOH (*n* = 1). Of two remaining patients with 12q CN-LOH, one carried an R392W polymorphism (rs770836648). This variant, which has a reported frequency of 0.000016 in normal populations [38], was also acquired in two patients with myeloproliferative disorders [39]. In our case, R392W was evidently of somatic origin because, although no remission sample was available, it was identified in 49% of reads, while allelic bias due to CN-LOH was 74%, as indicated by the read count for the rs3184504 C allele. Based on the crystal structure of the mouse SH2B1 SH2 domain [40], we constructed a model of the equivalent human SH2B3 sequence. This model strongly predicted that substitution of the aromatic ring of tryptophan for the amino group of the conserved 392 arginine residue would disrupt normal interaction with the autophosphorylation site of JAK2 (Fig. 3a–d) and likely equivalent sites on other kinases.

**Table 2:** Position and consequence of non-synonymous sequence changes identified in iAMP21-ALL patients with and without 12q CN-LOH and/or deletion of the SH2B3 region

Patient ID	12q CN-LOH	SH2B3 CN	Somatically acquired variants			Germ line variants	
			Nucleotide sequence change	Protein sequence change	Level	p.R262W AF C/T	Other variant (AF)
28	Yes	0	–			100/0%	
25	Yes	2	c.1174C > T	p.R392W^a^	49%	74/26%	
78	Yes	0^b^				93/7%	
44	Yes	2	c.1566dupC	p.E523Rfs	63%	100/0%	p.G451S (16%)
45	Yes	2				0/100%	
88	Yes	2	c.1198_1199insCT	p.E400Afs	69%	12/88%	
12	Yes	0	–			100/0%	
3	No	0	–			49/51%	
61	No	1	c.760T > G	p.C254G	52%	0/100%	
61	No	1	c.763_773del	p.SSIQ255-258Gfs	50%		
61	No	1	c.775_776delGA_insCG	p.E259R	51%		
19	No	2	–			100/0%	
75	No	2	–			100/0%	
77	No	2	–			100/0%	
81	No	2	–			100/0%	
84	No	2	–			0/100%	
76	No	2	–			0/100%	

^a^reported as a rare germline variant (rs770836648)

^b^Bi-allelic loss of start codon through exon 2 deletion. AF (Allelic Frequency) of R262W C / T alleles indicates homozygosity when AF are 0/100 or 100/0. In heterozygous patients allelic bias, caused by CN-LOH or deletion, is indicated by the ration of C and T alleles for the R262W variant

Lastly, we postulated a predisposing role for R262W, as re3184504 was homozygous for the T allele in patient 45, the only case where 12q CN-LOH but no *SH2B3* mutation or deletion was identified. We also noted that rs3184504 was never heterozygous in the six patients without 12q abnormalities. However, homozygosity of R262W in patient 45 was unrelated to 12q CN-LOH, as further sequencing demonstrated it was also homozygous in germline tissue and sequencing of 16 additional iAMP21-ALL cases identified the T/T, T/C and C/C genotypes in two, nine and five patients, respectively. Thus it is it unlikely that rs3184504 contributes to iAMP21-ALL and we saw no evidence for selection of other inherited *SH2B3* variants.

Collectively, CN and sequencing data linked 12q CN-LOH to bi-allelic loss of SH2B3 function, in all but one case (Fig. 3e) and, although rare in B-ALL overall, we reasoned that the incidence of *SH2B3* abnormalities was highly elevated in iAMP21-ALL. This finding prompted us to examine *SH2B3* abnormalities previously reported in B-ALL in the context of information relating to chromosome 21 CN status (Table 3). Remarkably, we discovered that in the two largest series described to date [28, 31], among 16 cases with *SH2B3* abnormalities, 12 carried previously unreported iAMP21/OSA21 and four had gain of WC21 (personal communications from M. Devidas on behalf of the Children’s Oncology Group, USA). In other studies [27, 29, 30, 32, 41], we found only a single example where chromosome 21 CN status was defined and an *SH2B3* abnormality was not associated with iAMP21/OSA21/wc21 gain. This case was also exceptional because an *SH2B3* mutation was inherited and unmasked by germline iCN-LOH [27].

**Table 3:** Chromosome 21 CN status in cases of B-ALL with abnormalities of *SH2B3* reported in the literature

Study	Diagnosis	patient ID	abnormality	Sub-type	chr 21 CN status
Roberts 2014 [28]	B-ALL	SJBALL021786	p.L224fs	Ph-like	+21
	B-ALL	SJBALL021415	Deletion	Ph-like	+21
	B-ALL	SJBALL020013	Deletion	Ph-like	iAMP21^a^
	B-ALL	SJHYPO018	p.V402M	Ph-like	+21
	B-ALL	SJBALL020984	p.L347fs	Ph-like	iAMP21^a^
	B-ALL	SJBALL239	N/A	Ph-like	iAMP21^a^
	B-ALL	SJBALL021720	p.A98D	Ph-like	iAMP21^a^
	B-ALL	SJBALL021058	p.S245_E3splice	Ph-like	iAMP21^a^
	B-ALL	SJBALL021373	p.R398fs	Ph-like	iAMP21^a^
Perez-Garcia 2013 [27]	B-ALL	NA	pD231fs	NA	Normal
	B-ALL	NA	pQ427fs	NA	NA
Lindqvist 2016 [30]	B-ALL	(B) ALL-128	p.L188P/p.Q191fs/ p.S192R/p.193_194del	Other	NA
	B-ALL	(B) ALL-109	p.G312V	t(12;21)	NA
Olsson 2015 [29]	B-ALL	2	Deletion	Other	NA
	B-ALL	5	Deletion	iAMP21	iAMP21
	B-ALL	20	fs^b^	HeH	+21
	B-ALL	N/A	p.S213R	NA	NA
	B-ALL	N/A	p.E78K	NA	NA
	B-ALL	N/A	p.R175W	NA	NA
	B-ALL	N/A	p.R397G	NA	NA
Ivanov Ofverholm 2016 [41]	B-ALL	KSALL12	Deletion	iAMP21	iAMP21
	B-ALL	KSALL20	Deletion	iAMP21	iAMP21
	B-ALL	KSALL44	Deletion	iAMP21	iAMP21
Reshmi 2017 [31]	B-ALL	PAVJMN	Missense^b^	Ph-like	iAMP21^a^
	B-ALL	PAUZPC	Deletion	Ph-like	iAMP21^a^
	B-ALL	PAVUJK	Deletion	Ph-like	iAMP21^a^
	B-ALL	PAUXDJ	fs^b^	Ph-like	iAMP21^a^
	B-ALL	PAWBPX	Deletion	Ph-like	iAMP21^a^
	B-ALL	PAVGVK	Deletion	Ph-like	iAMP21^a^
	B-ALL	PAVMFF	Deletion	Ph-like	+21, +21, +21^a^
Russell 2017 [32]	B-ALL	11706	fs^b^	iAMP21	iAMP21

^a^Chromosome CN status not disclosed on publication, personal communication from M. Devidas on behalf of the Childrens Oncology Group USA

^b^sequence change not presented

### STAT5 is activated by IL7 stimulation in iAMP21 cells with SH2B3 deletion

**Fig. 4:**
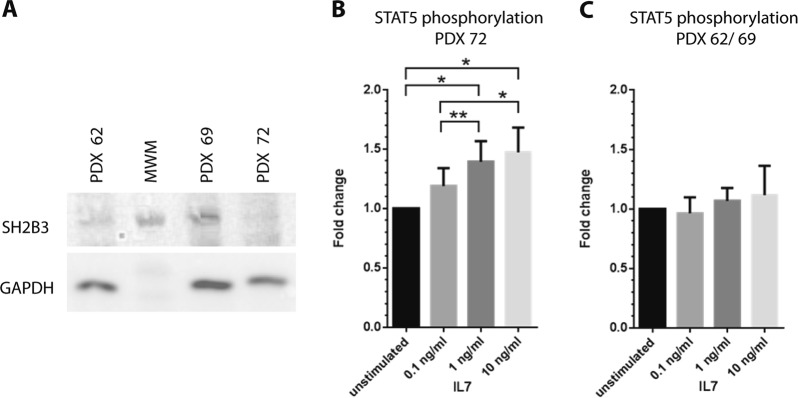
IL7 stimulates activation of STAT5 in iAMP21-ALL cells with SH2B3 deletion. **a** Immuno-blot with anti-SH2B3 confirmed loss of expression in PDX of patient 72 that carry a bi-allelic deletion of *SH2B3*. The full length protein was detected in patients 62 and 69 PDX that showed no evidence for *SH2B3* CN or coding sequence abnormalities. MWM (molecular weight marker) with 70 Kd band shown. **b** Treatment of PDX cells carrying *SH2B3* bi-allelic deletion with IL7 resulted in a dose related increase in STAT5 phosphorylation (four flow cytometric immunophenotyping experiments with cells from patient 72) *(*P* < 0.01) **(*P* < 0.05). **c** Analysis of PDX cells expressing wild-type SH2B3 failed to demonstrate any significant increase in STAT5 phosphorylation with IL7 treatment (four flow cytometric immunophenotyping experiments with cells from patient 62 [*n* = 2] and 69 [*n* = 2]). *P* values are for paired *t*-tests, error bars are standard error of the mean.

In two studies, *SH2B3* abnormalities co-occurred recurrently with potentially synergistic *IL7R* and/or *FLT3* activating mutations [28, 31], prompting us to investigate IL7R/JAK/STAT, FLT3/STAT and FLT3/RAS/RAF/ERK signalling pathways in iAMP21-ALL. PDX cells were used for this purpose as diagnostic patient cells were not available. We first demonstrated that SH2B3 protein was expressed in cells derived from two patients without a 12q abnormality, but not from cells derived from a patient carrying a bi-allelic deletion of *SH2B3*. On stimulation with IL7, we observed a clear dose dependent activation of STAT5, but not STAT1, 3, 4 or 6, in cells with SH2B3 inactivation. In contrast, IL7 stimulation of cells without evidence of SH2B3 inactivation showed no significant response to any STAT, while FLT3 ligand failed to activate either STATs or ERK1/2 in any of the cells tested (Fig. 4 and Supplementary Figure 3).

### 12q abnormalities may predict poor outcome in iAMP21-ALL

We were able to investigate the outcome of 26 iAMP21-ALL patients from this study who had been treated on UKALL97/99 (*n* = 5) or UKALL2003 (*n* = 21) clinical trials [1, 42]. After a median follow-up time of >5 years, there was evidence of an association with outcome: 5/7 (71%) patients with CN-LOH or deletion of 12q had relapsed and/or died compared with 4/19 (21%) patients without 12q abnormality (*p* = 0.03). Importantly, iAMP21 patients treated on ALL97/99 had a significantly higher rate of relapse compared with those treated on ALL2003 (70% vs. 16% at 5 years) due to the intensification of treatment received by these patients on UKALL2003 [1]. While prognosis overall of iAMP21 patients treated on ALL97 was worse than in UKALL 2003, there remained a strong association with 12q abnormality across both trials. This is illustrated by the fact that none of the three ALL 97 treated patients without 12q abnormality relapsed while 3/5 ALL 2003 patients with the abnormality relapsed/died.

## Discussion

Supporting some of our key findings, two previous reports noted an association between gross abnormalities of 12q affecting the *SH2B3* region and iAMP21-ALL [41, 43]. However, these studies were inconclusive as sequencing was not performed to confirm the involvement of *SH2B3*. Also the numbers of iAMP21-ALL cases analysed were insufficient to accurately assess the overall incidence, proportions affected by different types of lesions or influence on outcome. We studied a larger cohort of patients, in which we defined underlying *SH2B3* sequence abnormalities and performed preliminary analysis of outcome and also extended our investigation to include published data. We first established that in B-ALL, tCN-LOH 12q was exclusive to B-ALL with iAMP21/OSA21 or, at low incidence, with additional copies of wc21. Although existence of a second target for CN-LOH 12q cannot be formally ruled out, sequencing and copy number analysis identified bi-allelic inactivation of *SH2B3* as one consequence of these abnormalities. As further support for *SH2B3* as a relevant target, we showed the same pattern of high incidence in association with iAMP21-ALL and low incidence with aneuploidy 21 when SH2B3 was inactivated in the absence of CN-LOH 12q. Abnormality affecting *SH2B3* without any identifiable whole/partial chromosome 21 gain was extremely rare, with only a single case reported within the literature and a second identified through analysis of archival array data.

In our patients, all *SH2B3* lesions were bi-allelic, with CN-LOH being the dominant mechanism and deletion less frequently involved in conversion to homozygosity. We concluded that high copy number gain of specific sequences on chromosome 21, as seen in iAMP21-ALL, confers strong pressure for complete loss of SH2B3 function, while gain of one or two copies is associated with a similar though weaker selective environment. The absence of reported tCN-LOH 12q, in B-ALL without chromosome 21 gain, may be explained by the extreme rarity of *SH2B3* lesions among these cases. In haematological malignancies other than B-ALL, abnormalities of *SH2B3* have not been associated with 12q CN-LOH, suggesting less selective pressure for homozygous inactivation. Consistent with lineage specific tumour suppressor thresholds, *Tp53*-/- *SH2B3*± mice developed B-ALL in a manner that was strictly dependent on loss of the wild type *SH2B3* allele [44], while *SH2B3*± mice developed a milder form of the myeloproliferative disease seen in *SH2B3* -/- mice [45]. We also note that in B-ALL, *SH2B3* abnormalities were mostly whole gene deletions or truncating mutations, while in myeloid malignancies, substitutions affecting the PH domain predominated (reviewed in [35]).

SH2B3 is one of a three member family of SH2B adaptor proteins that share common motifs, including N-terminal dimerization, pleckstrin homology (PH) and Src homology 2 (SH2) domains [46]. Of the three, SH2B3 has the most prominent role in haematopoietic development, where it negatively regulates various receptor and non-receptor kinases, through direct interaction of the SH2 domain with phosphorylated tyrosine (reviewed in [47]). Critically, it functions in negative feedback loops controlling cell growth, development and survival signals because activated target kinases also induce *SH2B3* expression and activation through phosphorylation [44, 48, 49]. Our data clearly pose the question of why *SH2B3* loss of function should confer a strong selective advantage to B-ALL, specifically when certain chromosome 21 sequences are highly amplified through complex rearrangements. The mechanism by which iAMP21 promotes B-ALL is currently unknown and, in the absence of representative cell lines or genetically engineered animal models, challenging to investigate. Although usually defined at the cytogenetic level by ≥3 additional copies of *RUNX1*, there is little evidence that *RUNX1* overexpression drives iAMP21-ALL. Despite higher *RUNX1* copy number in iAMP21-ALL, it was not shown to be over-expressed relative to high hyperdiploid-ALL [23]. Furthermore B-ALL chromosome 21 copy number profiles are occasionally iAMP21-like, but not highly amplified for *RUNX1* compared with flanking sequences (Supplementary Figure 4). A second gene in the region, *DYRK1A* [3], is more consistently amplified and has been implicated in multiple signalling pathways contributing to cell growth and survival and cancer (reviewed in [50]). In common with B-ALL, the incidence of acute megakaryoblastic leukaemia (AMKL) is highly elevated in individuals with DS and *Dyrk1A* dosage increase was shown to co-operate with *Gata1* and *Mpl* mutations to promote AMKL [51]. Interestingly, although we found no examples of *SH2B3* abnormalities in DS-ALL, they were reported in DS-AMKL but not non-DS-AMKL [52, 53]. Therefore also in this highly specific haematological malignancy, *SH2B3* abnormalities may be preferentially selected on a background of chromosome 21 gain, in this instance with *DYRK1A* amplification demonstrated to be relevant. Although it has not been shown that *DYRK1A* amplification contributes to iAMP21-ALL, it does play a critical role in the large to small pre-B cell transition stage, where it induces quiescence through destabilisation of cyclin D3. However, while pre-B cells in Dyrk1a deficient mice had elevated levels of cyclin D3, they also displayed impaired proliferation, suggesting involvement in other, cyclin D3 independent, aspects of B-cell differentiation [54]. In iAMP21-ALL, co-operating abnormalities, such as loss of SH2B3 function, might therefore promote leukaemia by fine-tuning a mixture of oncogenic and tumour suppressor signals orchestrated by amplified *DYRK1A*. Like DYRK1A, GSK-3β is a multifunctional serine/threonine kinase implicated in diverse signalling pathways and both tumour promoting and tumour suppressor roles (reviewed in [55]). Interactions between GSK-3β and DYRK1A signalling almost certainly occur in in B cells; for instance GSK-3β also targets cyclin D for degradation [56] and is essential for the phosphorylation and nuclear exclusion of NFAT transcription factors, an event primed by DYRK1A and implicated as an important consequence of increased wc21 CN in DS-AMKL [51]. GSK-3β activity is regulated by posttranslational phosphorylation and in endothelial cells phosphorylation mediated by integrin was shown to depend on SH2B3 [57]. Loss of SH2B3 function could therefore modulate signals orchestrated by increased dose of DYRK1A through altered GSK-3β activity, although it remains to be determined whether this contributes to B-ALL.

Another likely arena for cooperation between abnormalities of chromosomes 21 and 12 involves the JAK/STAT pathway, because DYRK1A has been reported to phosphorylate STAT3 [58] and SH2B3 has been shown to negatively regulate JAK signalling [27, 44]. The only non-frameshift mutation identified among our cases was demonstrated, by homology modelling, to result in loss of interactions that repress activated JAK. Moreover, we found that iAMP21-ALL PDX cells with *SH2B3* bi-allelic deletions were more sensitive to IL7 induced STAT5 activation than those with wild type *SH2B3*. Evidence is also accumulating that in B-ALL, loss of SH2B3 function cooperates with abnormalities in addition to those of chromosome 21. Further implicating the JAK/STAT pathway, IL7R activating mutations were report in 8 of 16 cases with SH2B3 abnormalities [28, 31]. However the emerging picture is complex because other JAK-STAT pathway activating abnormalities appear to correlate negatively with *SH2B3* inactivation. For example JAK mutations were absent and only a single *CRLF2* activating rearrangement co-occurred with an *SH2B3* abnormality [28]. Among our iAMP21 patients tested for PAR1 deletions, that result in the *P2YR8-CRLF2* and consequent *CRLF2* over-expression, none were apparent in cases with (*n* = 7), but they were seen in 5 out of 26 cases (19%) without 12q abnormalities (data not shown). It may be that a background chromosome 21 gain promotes selection for activation of JAK/STAT, but not beyond a certain threshold. Cross talk between other pathways influenced by the various JAK/STAT activating rearrangements, may also favour selection of certain combinations but not others. SH2B3 is known to interact with other receptor and non-receptor kinases, such as FLT3 and LCK, that play a role in early B-cell development [49, 59]. We were unable to demonstrate ERK or STAT activation in *SH2B3* deleted PDX cells stimulated with FLT3 ligand nevertheless abnormalities activating FLT3 appear to be co-selected with those inactivating SH2B3 [28, 31] and a role in dysregulation of the RAS/RAF/MEK/ERK pathway should not be discounted. Although JAK-STAT and RAS pathway activation clearly contributes to iAMP21-ALL [11], these pathways are also commonly activated in patients without chromosome 21 abnormalities. The near-exclusive relationship we observed may therefore be driven by other SH2B3 interactions involving pathways dysregulated by high expression of *DYRK1A* and/or other genes amplified through iAMP21-ALL rearrangements. Notwithstanding these considerations, CN-LOH/deletion 12q may function as useful markers for cases that will potentially respond to JAK/STAT pathway inhibitors, indeed blast counts were reduced in PDX from one patient with IL7R activating mutation and SH2B3 deletion treated with ruxolitinib [33].

Importantly, we showed 12q abnormalities to be associated with an increased rate of treatment failure in iAMP21-ALL. Given the number of patients involved and level of significance, this finding is preliminary, but if confirmed in larger studies, may have important implications as iAMP21-ALL patients are classified as high risk [1] and currently at risk of the adverse consequences of intensive chemotherapy. Lastly further analysis of the tumour suppressor role of *SH2B3* in B-ALL may help to define critical signalling events elicited by increased doses of chromosome 21 gene expression in iAMP21-ALL, as well as other important sub-types with gain of wc21.

## Supplementary information

supplementary methods

supplementary table 1a

supplementary Table 1b

supplementary table 2

supplementary table 3

Supplementary table 4

Supplementary table 5

supplementary figures
