# Supplementary material for: SH2B3 inactivation through CN-LOH 12q is uniquely associated with B-cell precursor ALL with iAMP21 or other chromosome 21 gain
Source: Leukemia. 2019 Feb 28;33(8):1881–94. doi: 10.1038/s41375-019-0412-1 (PMC6756024; doi:10.1038/s41375-019-0412-1)
Supplement: Supplementary file 1 — supplementary methods [file 41375_2019_412_MOESM1_ESM.docx]

**Supplementary methods.**

**Methylation array analysis.** 1µg DNA from 14 iAMP21-ALL patients (seven with and seven without CN-LOH 12q) were analysed on Illumina HumanMethylation450 Bead Chips at the Wellcome Trust Clinical Research Facility, Edinburgh, UK. Array processing and quality control checks were performed as previously described.^1^ Using the R package minfi,^2^ genes in the 12q common region of CN-LOH predicted to be imprinted (*DCN, WIF1, FBRSL1* and *E2F2*)^3^ were investigated for differential methylation by CN-LOH 12q status and visualized using Gviz. Additionally, the entire region was investigated for CN-LOH dependent DMRs (differentially methylated regions) using DMRcate.^4^

**SNP 6.0 array analysis.** Arrays were prepared by the Paterson Institute Microarray Service (Manchester, UK) or AROS Applied Biotechnology AS (Aarhus, Denmark). Copy number and genotype analysis was performed manually using Genotyping Console version 4.1.4 (Affymetrix, Santa Clara, CA, USA). CN-LOH was defined as ≥ 1.5 Mb regions of loss of the AB genotype. An automated high throughput screen for CN abnormalities of the SH2B3 region was performed on arrays from 648 B-ALL patients deposited by Roberts *et al*.^5^ under European Genome-phenome Archive accession: EGAD00010000598. PennCNV-Affy^6, 7^ was used to normalise arrays and generate Log R ratio (LRR) data, DNAcopy^8, 9^ was used for segmentation along with custom R code for calling the *SH2B3* deletion events. Patients with called CN <1.7 within the *SH2B3* genomic region were analysed manually in Genotyping Console for CN abnormalities of chromosomes 12 and 21.

**Sequencing of *SH2B3* exons and breakpoints of an intragenic deletion.** To identify somatic and germline variants in *SH2B3,* coding exons (2-8) were individually amplified from 200 ng of patient DNA using primers and annealing temperatures as specified in Supplementary Table 5. First and second round PCR reactions were performed using FastStart High Fidelity polymerase (Roche Diagnostics, Mannheim, Germany) and standard conditions. Illumina adaptors and unique barcodes were added in the second round reactions using 1ul of a 1:100 dilution of the first round products, an annealing temperature of 60° and primers from the Access Array Barcode Library (Fluidigm, San Francisco, CA, USA). Second round amplification products were pooled at equimolar concentrations and sequenced on an Illumina MiSeq using 300bp paired-end chemistry (Edinburgh Genomics, University of Edinburgh, UK). Sequence reads were aligned to UCSC hg 19 and analysed as previously described.^10^ Called variants were annotated in ENSEMBL Variant Effect Predictor version 83^11^ to identify potential polymorphisms and all were confirmed by manual inspection in Integrated Genome Viewer.^12^

To confirm the presence of a focal bi-allelic exon 2 micro-deletion, identified by SNP 6.0 array analysis in patient 78, PCR was performed using combinations of primers pairs from introns 1 and 2 and FastStart High Fidelity polymerase according to the manufacturers recommendations. A product was amplified from the patient DNA, but not a wild type control using a single combination of primers (Supplementary table 5). The amplicon was Sanger sequenced (DBS Genomics, Durham University, Durham, UK) and the trace visualised in FinchTV (Geospiza, Inc, Seattle, WA, USA). Breakpoints were defined by aligning the amplicon sequence to the SH2B3 genomic sequence in BLAT.^13^ All genomic coordinates are according to GRCh37/hg19.

**SH2B3-SH2 domain homology model.** To predict the effect of the R392W variant on SH2B3 interaction with the JAK2 autophosphorylation site, we constructed a homology model based on the resolved co-crystal structure of the mouse SH2B1 SH2 domain (PDB code 2hdx) and an eleven residue phosphopeptide surrounding tyrosine pTyr813 within the activation loop of JAK2.^14^ A local Smith-Waterman alignment^15^ of 104 amino acids from SH2 domain sequences (human SH2B3 359-462 and mouse SH2B1 522-625) was performed using SSEARCH v36.^16^ A homology model of the human SH2B3-SH2 domain, based on sequence alignment and the published crystal structure of the mouse SH2B1 SH2 domain, was created using the Phyre2 server^17^ with 100% confidence. Superposition of mouse SH2B1-SH2 domain and the human SH2B3-SH2 homology model was performed using PDBeFOLD^18^ and resulted in a RMSD of 1.070Å.

**Western blotting.** Protein was extracted from PDX cells isolated from Mouse spleens and purified over Ficol. Whole cell lysates were prepared, fractionated and transferred according to standard procedures. Immunoblots were probed for SH2B3 and GAPDH with polyclonal Rabbit anti-SH2B3 (BS60245, Bioworld Technology, MN. USA) diluted 1:1000 and monoclonal mouse HRP conjugated anti-GAPDH diluted 1:15000 (ab9482, ABCAM. Cambridge, UK). Images were captured with a Synegene G:Box Chemi XL 1.4 (Cambridge, UK).

**STAT and ERK activation assays.** Stored viable leukaemia blasts isolated from PDX spleens and purified over FICOL were thawed quickly in RPMI containing 10% foetal bovine serum (FBS) (Gibco BRL), washed in RPMI medium and re-suspended in SFEM medium (Stemcell Technologies) containing 10% FBS at 10^6^ cells/ml before plating as 1ml aliquots into 12 well plates. Following one hour recovery individual wells were treated with 0, 0.1, 1.0 or 10ng/ml IL7 (Biolegends) or with 1.0, 10 or 100ng/ml Flt 3 ligand (Prospec Tamay) for 15 minutes at 37°C. Treated and control cells were washed in PBS, fixed according to manufacturer’s instructions with pre-warmed Lyse/Fix buffer (BD Biosciences, Oxford, UK) and permeabilised according to manufacturer’s instructions with Perm Buffer III (BD Biosciences, Oxford, UK). Stimulated cells were then re-suspended in 200 µl PBS and divided into two equal aliquots which were stained for 15 minutes at room temperature with fluorescently labelled antibodies; either pERK 1/2 (pT202/pY204) Alexa 647, or a mixture of STAT1 (pY701) BV421, STAT3 (pY705) PerCP Cy5.5, STAT4 (pY693) Alexa 647, STAT5 (pY694) PE Cy7 and STAT6 (pY641) PE (all from BD Biosciences, Oxford, UK). Unstimulated cells were re-suspended in 300 µl PBS and divided into three equal aliquots. One aliquot served as an unstained control and two were stained as above with the pERK or STAT antibodies. After two washes with 0.2% BSA in PBS, mean fluorescent intensity of each antigen was assessed using a BD Biosciences FACSCanto machine. Plots were created and significance of results calculated by paired T-tests using excel (Microsoft Corporation).

**Statistical analysis**. Significance of association between CN-LOH 12q/del 12q and iAMP21 was calculated by Fishers exact test. Patients treated on ALL97/99 and ALL2003 were followed-up as previously described^19^. Events were defined as failure to achieve a complete remission, relapse and death in remission. The frequency of events in different subgroups were compared using a Chi-squared test.

**References for supplementary methods**.

1. Gabriel AS, Lafta FM, Schwalbe EC, Nakjang S, Cockell SJ, Iliasova A*, et al.* Epigenetic landscape correlates with genetic subtype but does not predict outcome in childhood acute lymphoblastic leukemia. *Epigenetics* 2015; **10**(8)**:** 717-726.

2. Fortin JP, Triche TJ, Jr., Hansen KD. Preprocessing, normalization and integration of the Illumina HumanMethylationEPIC array with minfi. *Bioinformatics* 2017 Feb 15; **33**(4)**:** 558-560.

3. Morison IM, Ramsay JP, Spencer HG. A census of mammalian imprinting. *Trends Genet* 2005 Aug; **21**(8)**:** 457-465.

4. Peters TJ, Buckley MJ, Statham AL, Pidsley R, Samaras K, R VL*, et al.* De novo identification of differentially methylated regions in the human genome. *Epigenetics Chromatin* 2015; **8:** 6.

5. Roberts KG, Li Y, Payne-Turner D, Harvey RC, Yang YL, Pei D*, et al.* Targetable kinase-activating lesions in Ph-like acute lymphoblastic leukemia. *N Engl J Med* 2014 Sep 11; **371**(11)**:** 1005-1015.

6. Wang K, Li M, Hadley D, Liu R, Glessner J, Grant SF*, et al.* PennCNV: an integrated hidden Markov model designed for high-resolution copy number variation detection in whole-genome SNP genotyping data. *Genome Res* 2007 Nov; **17**(11)**:** 1665-1674.

7. Diskin SJ, Li M, Hou C, Yang S, Glessner J, Hakonarson H*, et al.* Adjustment of genomic waves in signal intensities from whole-genome SNP genotyping platforms. *Nucleic Acids Res* 2008 Nov; **36**(19)**:** e126.

8. Olshen AB, Venkatraman ES, Lucito R, Wigler M. Circular binary segmentation for the analysis of array-based DNA copy number data. *Biostatistics* 2004 Oct; **5**(4)**:** 557-572.

9. Venkatraman ES, Olshen AB. A faster circular binary segmentation algorithm for the analysis of array CGH data. *Bioinformatics* 2007 Mar 15; **23**(6)**:** 657-663.

10. Ryan SL, Matheson E, Grossmann V, Sinclair P, Bashton M, Schwab C*, et al.* The role of the RAS pathway in iAMP21-ALL. *Leukemia* 2016 Sep; **30**(9)**:** 1824-1831.

11. McLaren W, Gil L, Hunt SE, Riat HS, Ritchie GR, Thormann A*, et al.* The Ensembl Variant Effect Predictor. *Genome Biol* 2016 Jun 6; **17**(1)**:** 122.

12. Robinson JT, Thorvaldsdottir H, Wenger AM, Zehir A, Mesirov JP. Variant Review with the Integrative Genomics Viewer. *Cancer Res* 2017 Nov 1; **77**(21)**:** e31-e34.

13. Kent WJ. BLAT--the BLAST-like alignment tool. *Genome Res* 2002 Apr; **12**(4)**:** 656-664.

14. Hu J, Hubbard SR. Structural basis for phosphotyrosine recognition by the Src homology-2 domains of the adapter proteins SH2-B and APS. *J Mol Biol* 2006 Aug 04; **361**(1)**:** 69-79.

15. Smith TF, Waterman MS. Identification of common molecular subsequences. *J Mol Biol* 1981 Mar 25; **147**(1)**:** 195-197.

16. Pearson WR. Searching protein sequence libraries: comparison of the sensitivity and selectivity of the Smith-Waterman and FASTA algorithms. *Genomics* 1991 Nov; **11**(3)**:** 635-650.

17. Kelley LA, Mezulis S, Yates CM, Wass MN, Sternberg MJ. The Phyre2 web portal for protein modeling, prediction and analysis. *Nat Protoc* 2015 Jun; **10**(6)**:** 845-858.

18. Krissinel E, Henrick K. Secondary-structure matching (SSM), a new tool for fast protein structure alignment in three dimensions. *Acta Crystallogr D Biol Crystallogr* 2004 Dec; **60**(Pt 12 Pt 1)**:** 2256-2268.

19. Moorman AV, Robinson H, Schwab C, Richards SM, Hancock J, Mitchell CD*, et al.* Risk-directed treatment intensification significantly reduces the risk of relapse among children and adolescents with acute lymphoblastic leukemia and intrachromosomal amplification of chromosome 21: a comparison of the MRC ALL97/99 and UKALL2003 trials. *J Clin Oncol* 2013 Sep 20; **31**(27)**:** 3389-3396.
