# Supplementary material for: SH2B3 inactivation through CN-LOH 12q is uniquely associated with B-cell precursor ALL with iAMP21 or other chromosome 21 gain
Source: Leukemia. 2019 Feb 28;33(8):1881–94. doi: 10.1038/s41375-019-0412-1 (PMC6756024; doi:10.1038/s41375-019-0412-1)
Supplement: Supplementary file 8 — supplementary figures [file 41375_2019_412_MOESM8_ESM.docx]

**Supplementary Figures.
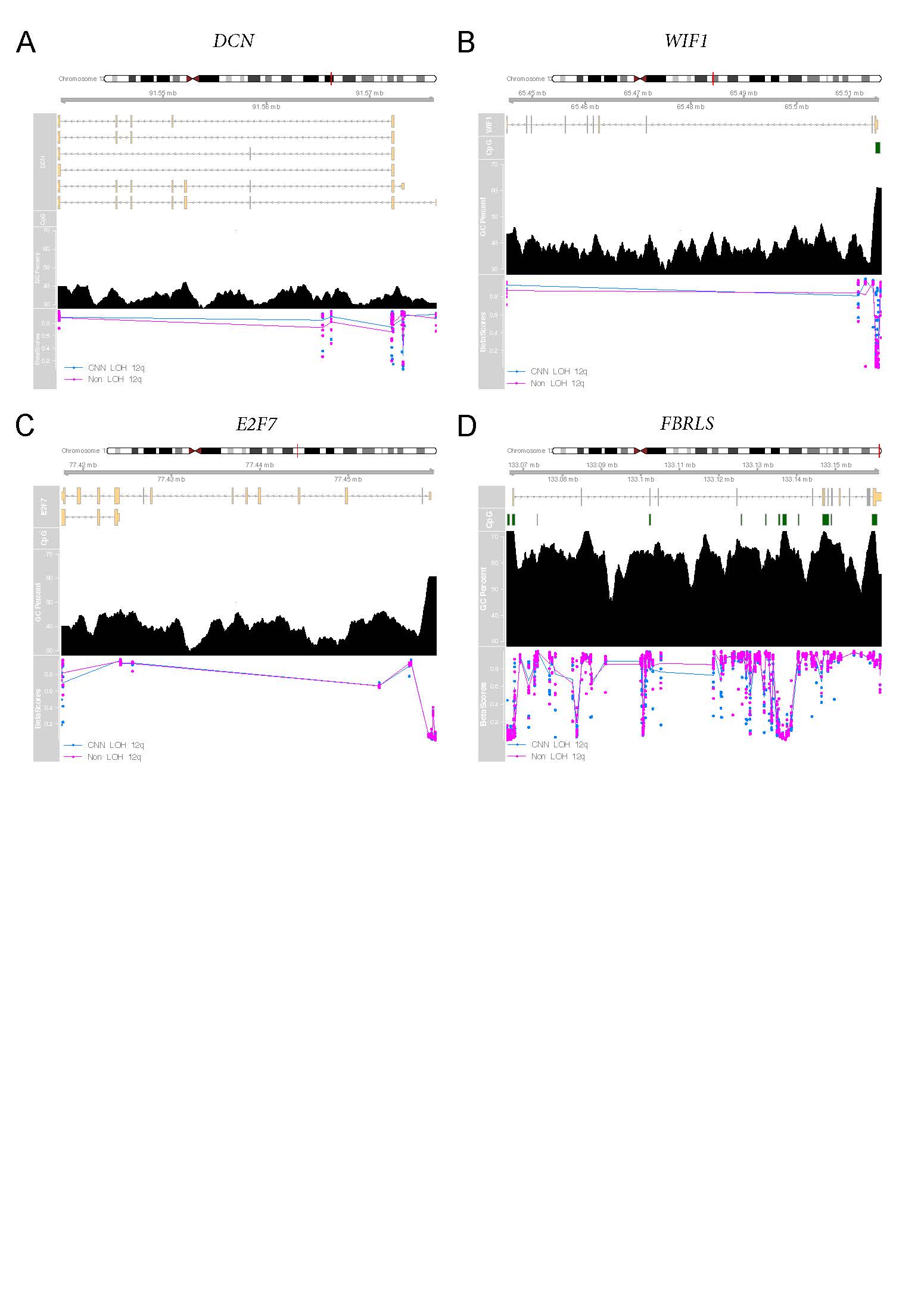
**

**Supplementary Figure 1**. **No LOH-dependent methylation differences in candidate genes on chromosome 12q**. Exon structure, CpG islands, GC content and proximal CpG probes are shown for candidate genes *DCN* (A), *WIF1* (B), *E2F7* (C) and *FBRSL1* (D). Individual methylation values are shown for non-LOH (purple) and CNN-LOH samples (blue). Average methylation of each group is shown as a line. Analysis of the entire 12q14-q telomeric region with DMRcate also failed to identify significant regions of differential methylation.


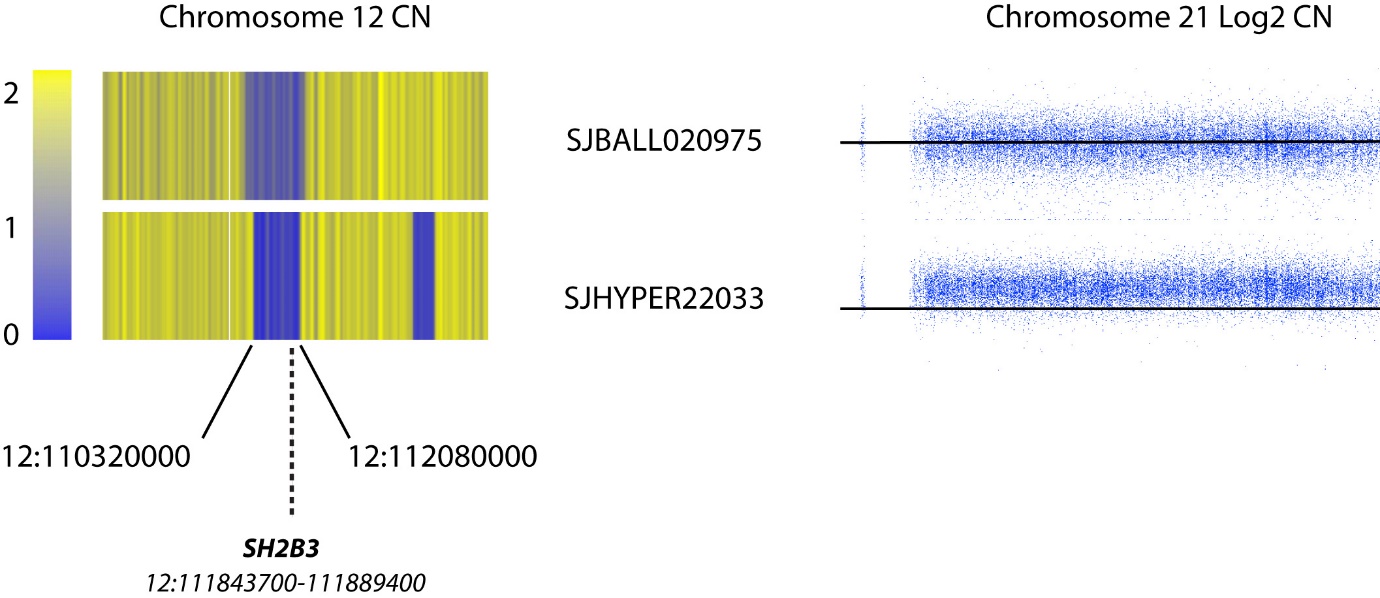


**Supplementary Figure 2**. Two focal deletions of the SH2B3 region identified through an automated screen of 648 B-ALL patients with publicly available SNP6.0 files. Heat maps, shown on the left, demonstrate mono-allelic deletion in both cases. Log 2 CN array profiles are shown on the right, with black lines indicating a CN of 2. For one patient (SJHYPER22033) the profile is consistent with two additional copies of whole chromosome 21 but in the second no evidence for aneuploidy or amplification of chromosome 21 is seen.

**
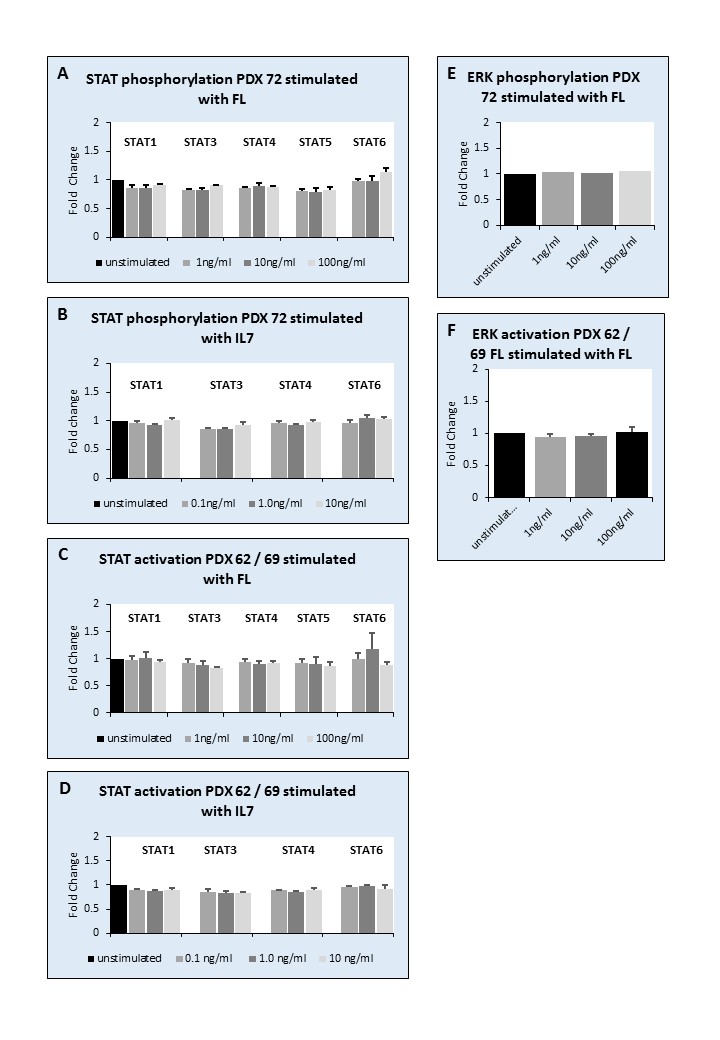
**

**Supplementary Figure 3. IL7 and FL fail to stimulate activation of STATS 1, 3, 4 and 6 or ERK in iAMP21-ALL PDX cells with or without deletion of SH2B3.** Treatment with 1, 10 or 100 ng/ml FLT3 ligand (FL) failed to induce phosphorylation of any STATs tested in PDX cells with deleted (patient 72) or wildtype (patients 62/69) *SH2B3*(**A** and **C**). Similarly no activation of STATs 1, 3, 4 and 6 were seen with 0.1, 1.0 or 10ng/ml IL7 treatment (**B** and **D**). This was in contrast to STAT5 which showed escalating levels of phosphorylation with the same IL7 treatments in cells with *SH2B3* deletion (Figure 4 main text). The same concentrations of FL also failed to increase levels of ERK 1/2 phosphorylation (**E** and **F**). All data presented are for flow cytometric immunophenotyping experiments. Error bars indicate standard error of the mean.


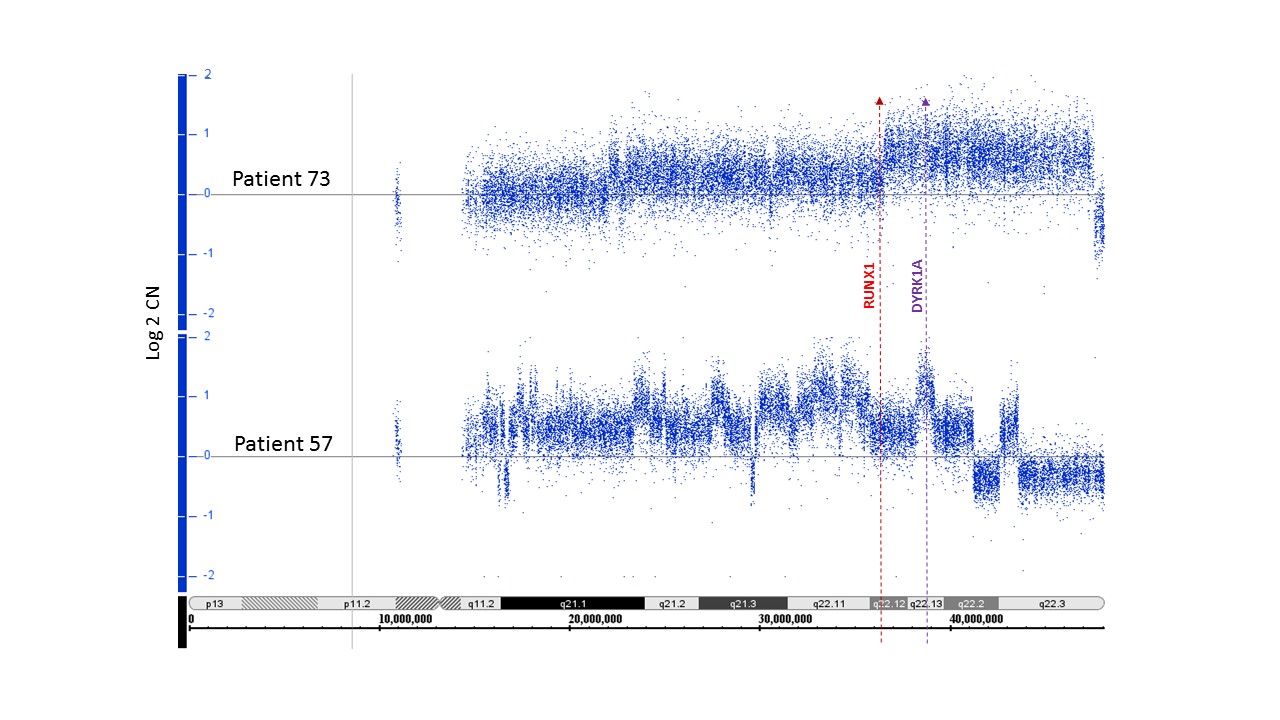


**Supplementary figure 4**. Examples of Log 2 chromosome 21 SNP6.0 CN profiles from iAMP21-ALL patients. In these cases iAMP21 was not identified by FISH as the genomic region containing *RUNX1* was amplified by only a single copy. The region containing DYRK1A was more highly amplified in both patients.
